# Physicians’ perspectives on estimating and communicating prognosis in palliative care: a cross-sectional survey

**DOI:** 10.3399/bjgpopen20X101078

**Published:** 2020-09-23

**Authors:** Marijanne Engel, Andrée van der Ark, Lia van Zuylen, Agnes van der Heide

**Affiliations:** 1 Department of Public Health, Erasmus MC, University Medical Center Rotterdam, Rotterdam, The Netherlands; 2 Department of Medical Oncology, Erasmus MC Cancer Institute, Rotterdam, The Netherlands

**Keywords:** communication, palliative care, prognosis, general practice, physicians

## Abstract

**Background:**

Advance care planning (ACP) can help to enhance the care of patients with limited life expectancy. Despite physicians’ key role in ACP, the ways in which physicians estimate and communicate prognosis can be improved.

**Aim:**

To determine how physicians in different care settings self-assess their performance in estimating and communicating prognosis to patients in palliative care, and how they perceive their communication with other physicians about patients’ poor prognosis.

**Design & setting:**

A survey study was performed among a random sample of GPs, hospital physicians (HPs), and nursing home physicians (NHPs) in the southwest of the Netherlands (*n* = 2212).

**Method:**

A questionnaire was developed that had three versions for GPs, HPs, and NHPs. Each specialism filled in an appropriate version.

**Results:**

A total of 547 physicians participated: 259 GPs, 205 HPs, and 83 NHPs. In the study, 61.1% of physicians indicated being able to adequately estimate whether a patient will die within 1 year, which was associated with use of the Surprise Question (odds ratio [OR] = 1.65, *P* = 0.042). In the case of a prognosis of <1 year, 75.0% of physicians indicated that they communicate with patients about preferences regarding treatment and care, which was associated with physicians being trained in palliative care (OR = 2.02, *P*=0.007). In cases where patients with poor prognosis are discharged after hospital admission, 83.4% of HPs indicated that they inform GPs about these patients’ preferences compared with 29.0% of GPs, and 21.7% of NHPs, who indicated that they are usually adequately informed about the preferences.

**Conclusion:**

The majority of physicians indicated that they believe they can adequately estimate patients’ limited life expectancy and that they discuss patients’ preferences for care. However, more physicians should be trained in communicating about patients’ poor prognosis and care preferences.

## How this fits in

In palliative care, ACP has been shown to improve concordance between patients’ own preferences and actual care, and to increase patient satisfaction with care. Timely ACP requires some estimate of patients’ life expectancy, but estimating and communicating patients’ poor prognosis can be complex. In this study, the majority of physicians indicated that they believe they can adequately estimate a patient’s limited life expectancy, but it was also found that communication about patients’ poor prognosis and related preferences can be improved, both with patients and with attending physicians in other care settings. More attention should be paid to training physicians in prognostic communication skills and coordination of roles and responsibilities related to ACP.

## Introduction

To improve the care of patients with a life-limiting disease, it is essential to identify their preferences with regard to medical treatment and care. This process of identifying goals and preferences is thought to prevent overtreatment as well as undertreatment, and is referred to as ACP.^1–3^ ACP has been shown to improve concordance between patients’ own preferences and actual care, and to increase patient satisfaction with care.^4,5^ A key element of ACP involves physicians estimating prognosis — especially in the case of poor prognosis — and communicating this to the patient, provided the patient is thought to be able to cope with such information.^3^ Indeed, research has shown that, to some degree, all patients with a life-limiting illness want to know about the course of their illness and their likely prognosis. Such information helps them indicate their preferences with regard to treatment and care.^2,4–7^

In daily practice, ACP is complex. Patients usually receive care from multiple healthcare professionals from different care settings^8^. Especially in the final months of their life, the majority of patients are transferred at least once between different care settings, which often involves an unplanned hospital admission.^9^ While estimating prognosis and discussing it with the patient are important at all stages of the illness trajectory of patients with a limited life expectancy,^10^ both HPs and non-HPs are engaged in this part of clinical practice.^10,11^ In the Netherlands, HPs, GPs, and NHPs have an important role in initiating or continuing palliative care.^11–13^ To ensure that patients’ needs are met and their preferences are honoured, physicians should also adequately communicate prognosis and related preferences to physicians from other care settings.

In palliative care, patients’ desire for information has been shown to contrast with a failure by physicians to predict prognosis.^12,14,15^ Studies have also shown that even if they are able to predict poor prognosis, GPs and HPs are reluctant to discuss poor prognosis and preferences regarding related treatment and care with patients and their relatives.^16–21^ Other studies have identified deficits in information exchange and communication between physicians, including those working in different care settings.^22–26^ Information exchange and communication are aspects of inter-organisational collaboration, which has been defined as *'* *a cooperative, inter-organisational relationship that is negotiated in an ongoing communicative process, and which relies on neither market nor hierarchical mechanisms of control* *'*.^27^

How physicians in different care settings self-assess their performance in estimating a poor prognosis and discussing it with patients has been poorly studied. Further, little attention has been paid to the experiences of physicians communicating about these issues with physicians in other care settings who are involved in the patient’s care. Therefore, the following research questions were studied:

1. How do physicians in different care settings self-assess their performance in estimating a poor prognosis and discussing it with patients in palliative care?
2. How do physicians in different care settings perceive their communication about patients’ poor prognosis and preferences regarding treatment and care with attending physicians working in other care settings?
3. In case of a poor prognosis, how do physicians assess the quality of collaboration with physicians working in other care settings?

## Method

### Study design

This cross-sectional survey study was part of a larger study on continuity in palliative care in the southwest region of the Netherlands. The study was performed among physicians working in primary care, hospitals, and nursing homes.

### Study population

The study population consisted of a random sample of physicians (*n* = 2212) from a full professional registry (IQVIA database OneKey), working in different care settings in the research region. GPs, HPs, and NHPs were included. Exclusion criteria were: junior doctors, and specialties that have relatively little to do with palliative care, such as ophthalmologists. A random stratified sample was taken; that is, 50% (*n* = 716) of all registered GPs, 50% (*n* = 1271) of all registered HPs from most specialties, and all (*n* = 225) registered NHPs. In July 2017, physicians were invited to fill in either a paper copy of the questionnaire or a digital version. Additionally, physicians were invited to participate in the study via institutes for training physicians in palliative care, and via professional newsletters.

### Questionnaire

A questionnaire was developed for this study with three versions (for GPs, HPs, and NHPs) to enable adequate formulation of the same questions and statements for each specialism. Questions were formulated based on earlier research about estimating prognosis^28,29^ and collaboration among HPs and GPs.^22^ Further, previously developed questionnaires were used.^30–32^

The general part of the questionnaire included questions on the responder's work setting, sex, age, practice experience (number of deceased patients in their practice per year), training in palliative care, self-reported use of the Surprise Question ('Would you be surprised if this patient would die in the next year?'),^14^ and degree of urbanisation of their work setting.

The questionnaire further focused on: 1) physicians' self-assessment of their performance in estimating prognosis (1 year, 3 months, 1 week) and communicating poor prognosis to patients; 2) communicating prognosis and related wishes for treatment and care with physicians from other care settings (HPs were asked for their communication with GPs, and GPs and NHPs were asked for their communication with HPs); and 3) perceived collaboration with physicians working in other care settings for patients with a poor prognosis in the past year (HPs were asked for their collaboration with GPs, and GPs and NHPs were asked for their collaboration with HPs). The questionnaire also contained open questions about experienced bottlenecks in collaboration with care providers from other organisations. Ten physicians tested a full draft of the questionnaire to assess the applicability (comprehension, formulation, and length of time). Their comments were incorporated in the final version of the questionnaire.

### Statistical analyses

The score on the numerical scale for quality of collaboration was categorised into 'inadequate' (scores ≤5) and 'adequate' (scores ≥6). In order to explore the potential association of responder characteristics with their self-reported performance in adequately estimating a prognosis of <1 year, a univariable regression analysis was performed. Those variables for which the association had a *P*<0.30 in the univariable analysis were entered in a multivariable analysis. Potential associations of responder characteristics with their self-reported performance in discussing wishes and expectations regarding treatment and care were explored similarly. Data were analysed using the statistical programme IBM SPSS Statistics (version 25). From the answers to open questions, after having coded them to themes, a few direct quotes were selected to illustrate the findings.

## Results

### Physician characteristics

The questionnaire was filled in by 547 physicians: 259 GPs (36.2% of GPs in sample), 205 HPs (16.1%), and 83 NHPs (36.9%). Of all responders, 51.7% indicated that they had ≥10 patients in their practice die per year. On the issue of training, 36.4% indicated that they had received extra training in the field of palliative care, ranging from any basic training after degree to specialist palliative care training. Of all responders, 19.7% reported use of the Surprise Question. Forty-seven per cent worked in a strongly urbanised area, 22.3% in a moderately urbanised area, and 24.9% in a rural area (Table 1).

**Table 1.** Characteristics of physicians

Characteristic		Physicians by care setting
Total,*N* = 547,*n* (%)	GP	HP	NHP^a^
*n* = 259,*n* (%)	*n* = 205,*n* (%)	*n* = 83,*n* (%)
Sex^b^	FemaleMale	256 (46.8)280 (51.2)	126 (48.6)131 (50.6)	89 (43.4)111 (54.1)	41 (49.4)38 (45.8)
Age, years ^c^	<4040–50>50	146 (26.7)155 (28.3)237 (43.3)	69 (26.6)75 (29.0)114 (44.0)	67 (32.7)62 (30.2)72 (35.1)	10 (12.0)18 (21.7)51 (61.4)
Number of patients die per year^d^	<55–1010–20>20	82 (15.0)163 (29.8)151 (27.6)132 (24.1)	27 (10.4)107 (41.3)84 (32.4)38 (14.7)	54 (26.3)45 (22.0)37 (18.0)58 (28.3)	1 (1.2)11 (13.3)30 (36.1)36 (43.4)
Training in the field of palliative care^d^	Extra training^e^No extra training or unknown	199 (36.4)329 (60.1)	118 (45.6)133 (51.4)	39 (19.0)159 (77.6)	42 (50.6)37 (44.6)
Self-reported use of Surprise Question^f,^ ^g^	Always or often^h^Sometimes or never	108 (19.7)400 (73.1)	59 (22.8)192 (74.1)	32 (15.6)154 (75.1)	17 (20.5)54 (65.1)
Degree of urbanisation work setting^i^	Extremely or strongly urbanised ^j^Moderately urbanisedHardly or not urbanised	257 (47.0)122 (22.3)136 (24.9)	96 (37.1)49 (18.9)112 (43.2)	126 (61.5)47 (22.9)6 (2.9)	35 (42.2)26 (31.3)18 (21.7)

^a^Nursing homes in the Netherlands are facilities for vulnerable older and other persons, where medical care is provided by on-site nursing home physicians.^44^ ^b^Missing: *n* = 11 (2.0%). ^c^Missing: *n* = 9 (1.6%). ^d^Missing: *n* = 19 (3.5%). ^e^Responder is specialist in palliative care by ‘specialist palliative care’ education, is working as a hospice physician, or reports other expertise in palliative care. ^f^‘Surprise Question’: Would you be surprised if this patient would die in the next year? ^g^Missing: *n* = 39 (7.1%). ^h^Physicians could answer on a four-point scale: ‘always’, ‘often’, ‘sometimes’, ‘never’. ‘Always’ or ‘often’, and ‘sometimes’ or ‘never’ answers were combined in this table. ^i^Missing: *n* = 32 (5.9%). ^j^Degree of urbanisation is based on ZIP code of work setting, related to environmental address density;^45^ 1) extremely urbanised (environmental address density of ≥2500 addresses per km^2^); 2) strongly urbanised (1500–2500 addresses per km^2^); 3) moderately urbanised (1000–1500 addresses per km^2^); 4) hardly urbanised (500–1000 addresses per km^2^); 5) not urbanised (<500 addresses per km^2^). HP = hospital physician. NHP = nursing home physician.

### Estimating and communicating prognosis with patient

Of all physicians, 61.1% stated that they can ‘always’ or ‘often’ adequately estimate if a patient will die within 1 year. When a patient is estimated to have a prognosis of <1 year, the majority of all physicians (75.0%) indicated that they ‘always’ or ‘often’ will discuss with the patient his or her wishes and expectations regarding treatment and care. On the issue of whether the physician would have a conversation with the patient about his or her worries, psychosocial aspects, and/or meaning of life questions, 43.4% of HPs answered either ‘always’ or ‘often’, compared with 73.0% of GPs and 71.1% of NHPs (Table 2). In univariable regression analysis, it was found that self-reported use of the Surprise Question had a statistically significant association with physicians’ self-reported performance in adequately estimating a prognosis of <1 year (*P* = 0.025). Based on *P*<0.30 in univariate analysis, age (*P* = 0.188), practice experience (*P* = 0.071), and self-reported use of the Surprise Question (*P* = 0.025) were included in the multivariable analysis. A statistically significant association was only found with use of the Surprise Question (OR = 1.65, 95% confidence interval [CI] = 1.018 to 2.688, *P* = 0.042). It was also found that both training in palliative care (*P* = 0.001) and self-reported use of the Surprise Question (*P* = 0.027) were statistically significantly associated with physicians’ self-reported performance in discussing wishes and expectations regarding treatment and care with the patient in the case that a patient had a prognosis of <1 year. In a multivariable analysis, a statistically significant association was only found with training in palliative care (OR = 2.02, 95% CI = 1.210 to 3.384, *P* = 0.007).

**Table 2.** Estimating and communicating prognosis with the patient

Statement		Physicians by care setting
Total,*N* = 547,*n* (%)	GP,*n* = 259,*n* (%)	HP,*n* = 205,*n* (%)	NHP,*n* = 83,*n* (%)
I can adequately estimate if a patient will die within a year ^a,b^	334 (61.1)	163 (62.9)	121 (59.0)	50 (60.2)
I think it is important to consider whether a patient will possibly die within a year^c^	416 (76.1)	197 (76.1)	157 (76.6)	62 (74.7)
If I expect a patient to die within a year, I will discuss his or her wishes and expectations regarding treatment and care^d^	410 (75.0)	197 (76.1)	147 (71.7)	66 (79.5)
If I expect a patient to die within a year, I will have a conversation about his or her worries, psychosocial aspects, and/or meaning of life questions^e^	337 (61.6)	189 (73.0)	89 (43.4)	59 (71.1)
I can adequately estimate if a patient will die within 3 months^f^	365 (66.7)	188 (72.6)	127 (62.0)	50 (60.2)
I think it is important to consider whether a patient will possibly die within 3 months^g^	507 (92.7)	253 (97.7)	180 (87.8)	74 (89.2)
If I expect a patient to die within 3 months, I will discuss his or her wishes and expectations regarding treatment and care^h^	501 (91.6)	249 (96.1)	176 (85.9)	76 (91.6)
If I expect a patient to die within 3 months, I will have a conversation about his or her worries, psychosocial aspects and/or meaning of life questions^i^	444 (81.2)	246 (95.0)	130 (63.4)	68 (81.9)
I can adequately estimate if a patient will die within a week^j^	416 (76.1)	206 (79.5)	145 (70.7)	65 (78.3)
I think it is important to consider whether a patient will possibly die within a week^k^	512 (93.6)	250 (96.5)	187 (91.2)	75 (90.4)
If I expect a patient to die within a week, I will discuss his or her wishes and expectations regarding treatment and care^l^	505 (92.3)	249 (96.1)	180 (87.8)	76 (91.6)
If I expect a patient to die within a week, I will have a conversation about his or her worries, psychosocial aspects and/or meaning of life questions^m^	469 (85.7)	240 (92.7)	159 (77.6)	70 (84.3)

^a^Physicians could answer on a four-point scale: ‘always’, ‘often’, ‘sometimes’, ‘never’. Only combined ‘always’ or ‘often’ answers are shown. ^b^Missing total: *n* = 21 (3.8%) (GP: *n* = 6, HP: *n* = 8, NHP: *n* = 7). ^c^Missing total: *n* = 15 (2.7%) (GP: *n* = 1, HP: *n* = 6, NHP: *n* = 8). ^d^Missing total: *n* = 20 (3.7%) (GP: *n* = 3, HP: *n* = 10, NHP: *n* = 7). ^e^Missing total: *n* = 25 (4.6%) (GP: *n* = 7, HP: *n* = 10, NHP: *n* = 8). ^f^Missing total: *n* = 22 (4.0%) (GP: *n* = 6, HP: *n* = 8, NHP: *n* = 8). ^g^Missing total: *n* = 17 (3.1%) (GP: *n* = 3, HP: *n* = 7, NHP: *n* = 7). ^h^Missing total: *n* = 23 (4.2%) (GP: *n* = 5, HP: *n* = 11, NHP: *n* = 7). ^i^Missing total: *n* = 21 (3.8%) (GP: *n* = 3, HP: *n* = 11, NHP: *n* = 7). ^j^Missing total: *n* = 23 (4.2%) (GP: *n* = 6, HP: *n* = 10, NHP: *n* = 7). ^k^Missing total: *n* = 17 (3.1%) (GP: *n* = 1, HP: *n* = 9, NHP: *n* = 7). ^l^Missing total: *n* = 21 (3.8%) (GP: *n* = 3, HP: *n* = 11, NHP: *n* = 7). ^m^Missing total: *n* = 26 (4.8%) (GP: *n* = 6, HP: *n* = 13, NHP: *n* = 7). HP = hospital physician. NHP = nursing home physician.

When a patient is estimated to have a prognosis of <3 months or <1 week, 66.7% and 76.1% of all physicians respectively stated that they can ‘always’ or ‘often’ adequately estimate this. More than nine in 10 physicians will then ‘always’ or ‘often’ discuss with the patient his or her wishes and expectations regarding treatment and care. (Table 2).

### Communicating prognosis and collaboration with physicians working in other care settings

In cases where it is determined in the hospital that a patient has a serious incurable disease, 94.1% of the HPs indicated that they would ‘always’ or ‘often’ inform the GP. The study found 86.1% of GPs and 59.0% of NHPs indicated that they have ‘always’ or ‘often’ been adequately informed about this by HPs (Table 3).

**Table 3.** Communicating prognosis with physicians working in other care settings, and perceived quality of collaboration

Statement (GP/NHP or HP variant)	Physicians by care setting
GP,*n* = 259,*n* (%)	HP,*n* = 205,*n* (%)	NHP,*n* = 83,*n* (%)
GP/NHP: In case it is determined in the hospital that a patient has a serious incurable disease, I am adequately informed about this.^a,b^HP: In case it is determined in the hospital that a patient has a serious incurable disease, I inform the GP adequately.		223 (86.1)	193 (94.1)	49 (59.0)
GP/NHP: Collaboration with care providers from the hospital is important for me to be able to provide good care.^c^HP: Collaboration with the GP is important for me to be able to provide good care.		233 (90.0)	190 (92.7)	74 (89.2)
GP/NHP: In case a patient known to have a limited life expectancy is admitted to hospital unscheduled, I am informed about this within 48 hours.^d^HP: In case a patient known to have a limited life expectancy is admitted to hospital unscheduled, I inform the GP adequately.		187 (72.2)	127 (62.0)	29 (34.9)
GP/NHP: In case a patient with a limited life expectancy is discharged after being admitted to hospital under my responsibility, I am informed about this within 24 hours.^e^HP: In case a patient with a limited life expectancy is discharged after being admitted to hospital under my responsibility, I inform the GP adequately.		186 (71.8)	187 (91.2)	55 (66.3)
GP/NHP: In case a patient with a limited life expectancy is discharged after an unplanned admission to hospital, I (GP or NHP) receive adequate information from the hospital about^f^:HP: In case a patient with a limited life expectancy is discharged after an unplanned admission to hospital, I (HP) inform the GP adequately about:				
… the medical situation of the patient		217 (83.8)	193 (94.1)	52 (62.7)
… the psychosocial situation of the patient		39 (15.1)	127 (62.0)	11 (13.3)
… the prognosis of the patient		113 (43.6)	158 (77.1)	24 (28.9)
… the medication that patient uses		221 (85.3)	187 (91.2)	77 (92.8)
… wishes and agreements of or with patient about treatment and care		75 (29.0)	171 (83.4)	18 (21.7)
GP/NHP: In case a patient dies in hospital during admission, I am informed.^g^HP: In case a patient dies in hospital during admission, I inform the GP about this within 24 hours.		223 (86.1)	187 (91.2)	26 (31.3)
GP/NHP: In case a patient dies outside the hospital, I ensure that the treating HP is informed about this.^h^HP: In case a patient dies outside the hospital, I am informed about this.		142 (54.8)	31 (15.1)	40 (48.2)
GP/NHP: Poor collaboration with the HP hinders me in providing good care to patients with a limited life expectancy.^i^HP: Poor collaboration with the GP hinders me in providing good care to patients with a limited life expectancy.		86 (33.2)	41 (20.0)	40 (48.2)
Quality of collaboration with the hospital (was asked to GP and NHP) or with the GP (was asked to HP) for patients with a limited life expectancy in the past year.^j^	Mean (SD)^k^Inadequate (≤5)Adequate (≥6)	7.3 (1.0)10 (3.9)245 (94.6)	7.5 (1.1)6 (2.9)190 (92.7)	6.2 (1.3)21 (25.3)62 (74.7)

^a^Physicians could answer on a four point scale: ‘always’, ‘often’, ‘sometimes’, ‘never’. Only combined ‘always’ or ‘often’ answers are shown. ^b^Missing total: *n* = 14 (2.6%) (GP: *n* = 4, HP: n = *4*, NHP: *n* = 6). ^c^Missing total: *n* = 9 (1.6%) (GP: *n* = 5, HP: *n* = 4, NHP: *n* = 0). ^d^Missing total: *n* = 22 (4.0%) (GP: *n* = 5, HP: *n* = 10 , NHP: *n* = 7). ^e^Missing total: *n* = 25 (4.6%) (GP: *n* = 8, HP: *n* = 10, NHP: *n* = 7). ^f^Missing total for ‘...the medical situation of the patient’: *n* = 14 (2.6%) (GP: *n* = 4, HP: *n* = 10, NHP: *n* = 0). ‘...the psychosocial situation of the patient’: *n* = 13 (2.4%) (GP: *n* = 2, HP: *n* = 11, NHP: *n* = 0). ‘… the prognosis of the patient’: *n* = 20 (3.7%) (GP: *n* = 5, HP: *n* = 15, NHP: *n* = 0). ‘... the medication that patient uses’: *n* = 15 (2.7%) (GP: *n* = 4, HP: *n* = 11, NHP: *n* = 0). ‘… wishes and agreements of/with patient about treatment and care’: *n* = 19 (3.5%) (GP: *n* = 7, HP: *n* = 10, NHP: *n* = 2). ^g^Missing total: *n* = 24 (4.4%) (GP: *n* = 6, HP: *n* = 11, NHP: *n* = 7). ^h^Missing total: *n* = 19 (3.5%) (GP: *n* = 2, HP: *n* = 10, NHP: *n* = 7). ^i^Missing total: *n* = 12 (2.2%) (GP: *n* = 4, HP: *n* = 8, NHP: *n* = 0). ^j^Missing total: *n* = 13 (2.4%) (GP: *n* = 4, HP: *n* = 9, NHP: *n* = 0). ^k^Physicians were asked to give a score for the quality of collaboration with physicians from outside their own organisation on a scale from 1 to 10 with a higher score representing a higher assessment of quality; an inadequate score was ≤5, an adequate score was ≥6. HP = hospital physician. NHP = nursing home physician. SD = standard deviation.

In cases where a patient with a limited life expectancy is discharged after an unplanned admission to the hospital, 77.1% of HPs indicated that they ‘always’ or ‘often’ inform the GP about the prognosis of the patient, compared with 43.6% of GPs and 28.9% of NHPs who indicated that they are ‘always’ or ‘often’ adequately informed about this. With regard to wishes and agreements of or with the patient about treatment and care, 83.4% of HPs indicated that they ‘always’ or ‘often’ adequately inform the GP about this, compared with 29.0% of GPs and 21.7% of NHPs who indicated that they are ‘always’ or ‘often’ adequately informed about this (Table 3).

Physicians’ mean score for quality of their collaboration with physicians from other care settings with regard to care for patients with a limited life expectancy was 7.2 (standard deviation [SD] 1.2). NHPs had the lowest mean score (6.2) and HPs the highest mean score (7.5). Of all physicians, 30.5% indicated that poor collaboration with physicians from other care settings ‘always’ or ‘often’ hinders them in providing adequate care to patients with a limited life expectancy (Table 3).

In answers to open questions, 421 of 547 responders reported ≥1 bottlenecks in their collaboration with care physicians from other care settings; 34 physicians mentioned that they experienced few or no bottlenecks. If physicians reported bottlenecks, the most mentioned themes were communication and exchange of information (Table 4).

**Table 4.** Open answers: experienced bottlenecks in collaboration between GPs/NHPs (their perspectives) and HPs, and between HPs (their perspectives) and GPs^a^

	Physicians by care setting
GP, *n* = 259	HP, *n* = 205	NHP, *n* = 83
Communication and/or consultation, *n*	120Quote:*'Too little communication, would like to be called by hospital about diagnosis, discharge, prognosis etc, now I know sometimes nothing, neither about the wishes of the patient than I know nothing.'* (GP 95)	98Quote:*'Especially the difficult accessibility of general practitioners both in and outside working hours is a problem. In addition, not all details of the GP are known and readily available.'* (HP 116)	47Quote:*'Communication about the seriousness of the situation and limited treatment possibilities is sometimes not provided. The nursing home must then still bring the bad news.'* (NHP 82)
Exchange of information, *n*	102Quote:*'Too late information: patient is with me after hospital visit without me being informed, and wants to talk to me about decisions to be made. Especially a problem in case of interim changes.'* (GP 31)	21Quote:*'Not always feedback from a general practitioner when care is transferred, only then suddenly contact again in an emergency situation.'* (HP 122)	46Quote:*'Rarely enough information about the patient’s wishes around end of life.'* (NHP 66)
No or few bottlenecks, *n*	15	17	2

^a^421 responders reported ≥1 bottlenecks in collaboration with care physicians from other care settings. Open answers were coded to themes, with a maximum of four themes for one answer. Some examples of quotes are given in the table. HP = hospital physician. NHP = nursing home physician.

## Discussion

### Summary

The results of this study suggest that the majority of physicians believe they can adequately estimate a patient’s limited life expectancy, and, in cases where prognosis is poor, discuss the patient's wishes with the patient. However, this study also suggests that information transfer and communication concerning patients’ wishes for treatment and care can be improved. Multivariable analysis showed a statistically significant association between use of the Surprise Question and physicians’ self-reported performance in adequately estimating a prognosis of <1 year, and between training in palliative care and self-reported performance in discussing preferences regarding treatment and care in case where a patient has a prognosis of <1 year.

### Strengths and limitations

A strength of the study is that a random sample was surveyed from a professional registry of GPs, HPs, and NHPs in the research region. A limitation is that the questions about estimating and communicating prognosis were somewhat general, which may have resulted in the physicians’ responses representing their views on the subject rather than their actual behaviour. It is expected that the findings can be generalisable to other parts of the Netherlands and Europe, although caution is advised because of differences in healthcare systems and in the education of care providers. Finally, the cross-sectional nature of the study limits the possibility of making robust causal inferences.

### Comparison with existing literature

A significant association was found between use of the Surprise Question and physicians’ self-reported performance in adequately estimating a prognosis of <1 year. In general, the process of estimating prognosis and communicating this to patients has been found to be complex. Studying nuances of this process appears to be difficult given the great diversity in patients and disorders, and in care settings.^10,21^ In a systematic review of predictions of survival in palliative care, White *et al* found no subgroup of physicians that consistently performed better in estimating prognosis.^10^ The finding that physicians using the Surprise Question reported better performance in adequately estimating a prognosis of <1 year, supports studies that mention the Surprise Question as a simple and feasible tool helping physicians to adequately identify patients with palliative care needs.^14,15,33^ Nevertheless, the Surprise Question has a rather low specificity and positive predictive value, which means that many patients unexpectedly live longer than 1 year.^15,33^ The main purpose of estimating patients’ prognosis is not necessarily to inform the patient about their estimated life expectancy in years, months, or weeks. Rather, the emphasis is on communicating with the patient about their deteriorating situation.^3,14,34^ Nevertheless, in cases where a physician expects a patient to die within a year, 21.4% of the physicians surveyed here indicated that they would not talk to the patient about their wishes and expectations. Possible explanations for this finding are that these physicians consider it the role of an attending physician working in another care setting,^11^ that physicians feel that the patient cannot cope with such information,^19,20^ or restraint on the part of physicians regarding the value of ACP.^11,35^ The finding could also be related to the healthcare reimbursement system in the Netherlands: only since 2018 can HPs request reimbursement for an *‘* *e* *xtensive consultation for careful consideration of treatment options, together with the patient and/or his/her representative* *’* (translated from Dutch).^36^

On the other hand, a significant association was found between being trained in palliative care and discussing preferences regarding treatment and care in the case that a patient has a prognosis of <1 year. This finding supports findings from other studies.^13,21^ Thoonsen *et al* found that 1 year after the start of a training programme on how to provide structured anticipatory palliative care, GPs performed significantly better in estimating a limited life expectancy and in providing multidimensional care.^13^ In a review and synthesis of best practices in communication about serious illness care goals, Bernacki *et al* found that training of HPs is one of the most promising interventions to promote conversations with patients about preferences regarding end-of-life care.^21^

In general, when physicians were asked about their collaborations with physicians from other care settings for patients with a limited life expectancy in the past year, they were moderately positive, with HPs giving the highest mean score for the quality of collaboration, and NHPs the lowest. Considerable differences were found in experiences between the hospital and non-hospital physicians: while HPs often stated that they adequately inform the GP about patients with a limited life expectancy, GPs and NHPs often indicated that they are not adequately informed. It was also found that, in the experience of GPs and NHPs, the handover from the HP often lacks information regarding prognosis and patients’ wishes for treatment and care. Other studies have also found such deficits.^8,37–39^ With regard to palliative care, den Herder-van der Eerden *et al* ^8^ and Seamark *et al* ^39^ concluded that the information exchange between healthcare providers from different care settings in palliative care is relatively poor.

There are a number of explanations for the differences that were found between physicians. First, HPs may overestimate the frequency and content of their own communication with patients. A second explanation could be that HPs do not document this information adequately. Other studies found that around 30% of treatment and care preferences — as expressed by the patient — were documented in the medical record.^17,40^ This indicates that the proportion of HPs in the study who communicate with patients about poor prognosis and related preferences is probably higher than the proportion who document this adequately in the medical record. This lack of documentation probably leads to inadequate information in the medical handover.

A third possible explanation is that it is unclear for the HP exactly when patients’ wishes and expectations should be communicated, and to what extent this is part of their role and responsibilities.^11,17^ This may have to do with differences in professionalisation regarding ACP. Professionalisation is described as a process that serves to secure and protect exclusive areas of knowledge, skills, and expertise of professionals in the healthcare system.^41^ This means that physicians in different care settings have different professional values and follow different procedures.^27,41,42^ Other studies have found differences in terminology and in attitudes towards palliative care and ACP between physicians working in different care settings.^28,40,43^

### Implications for practice

In conclusion, the findings suggest that more shared professionalisation towards ACP and communicating prognosis in palliative care may facilitate collaborative partnership in ACP between physicians working in different care settings. To achieve this, first, professional physicians’ associations should, in mutual consultation, give direction to the coordination of roles and responsibilities related to ACP. Second, education and training in practice require more attention for communicating poor prognosis and related preferences with patients and with other physicians.
